# Quantifying the effect of genetic, environmental and individual demographic stochastic variability for population dynamics in *Plantago lanceolata*

**DOI:** 10.1038/s41598-021-02468-9

**Published:** 2021-11-30

**Authors:** Ulrich K. Steiner, Shripad Tuljapurkar, Deborah A. Roach

**Affiliations:** 1 Institute of Biology, Freie Universität Berlin, Berlin, Germany; 2 Department of Biology, Stanford University, Stanford, CA USA; 3 Department of Biology, University of Virginia, Charlottesville, VA USA

**Keywords:** Ecological modelling, Evolutionary ecology, Population dynamics, Theoretical ecology

## Abstract

Simple demographic events, the survival and reproduction of individuals, drive population dynamics. These demographic events are influenced by genetic and environmental parameters, and are the focus of many evolutionary and ecological investigations that aim to predict and understand population change. However, such a focus often neglects the stochastic events that individuals experience throughout their lives. These stochastic events also influence survival and reproduction and thereby evolutionary and ecological dynamics. Here, we illustrate the influence of such non-selective demographic variability on population dynamics using population projection models of an experimental population of *Plantago lanceolata.* Our analysis shows that the variability in survival and reproduction among individuals is largely due to demographic stochastic variation with only modest effects of differences in environment, genes, and their interaction. Common expectations of population growth, based on expected lifetime reproduction and generation time, can be misleading when demographic stochastic variation is large. Large demographic stochastic variation exhibited within genotypes can lower population growth and slow evolutionary adaptive dynamics. Our results accompany recent investigations that call for more focus on stochastic variation in fitness components, such as survival, reproduction, and functional traits, rather than dismissal of this variation as uninformative noise.

## Introduction

Population dynamics are driven by simple demographic events, the survival and reproduction of individuals in a population^1^. To understand and forecast such dynamics, it is important to quantify the sources of variability in demographic fates. Individual variability in survival and reproduction has been ascribed to genetic variability, environmental stochasticity, and demographic stochasticity^2–4^, but these causes of variation may be selective or non-selective (i.e., neutral)^5^. “Genetic variability” includes additive genetic variance, non-additive genetic variance (dominance and epistasis) and genotype-environment interactions (phenotypic plasticity). “Environmental stochasticity” usually includes temporal fluctuations affecting all individuals in a population, but might not include differences below the population level, e.g., as caused by temporal or spatial environmental differences within the population^2,6^. Additionally, where genotypes are not known, environmental stochasticity frequently includes genotype-environment interactions^2^. “Demographic stochasticity” is defined by Lande et al. (2003) and Melbourne and Hastings (2008) as the result of independent chance events, e.g., individuals with identical reproductive and survival rates may differ in how many offspring they produce or how long they live. Demographic stochasticity has also been used to describe demographic heterogeneity, when individuals have distinct probability distributions for reproduction and survival^6^. Such differences can be fixed at birth or can change dynamically during the life course^7^, and may in turn be influenced by genetic differences, maternal differences or chance^6^. The amount of demographic stochasticity can be estimated by calculating demographic variance^8^, a quantity that has earlier been called individual stochasticity^9^, and dynamic heterogeneity^7,10^. In spite of these ambiguities, we know that including or excluding different types of variation, and not accurately differentiating among selective and non-selective causes of variation, can substantially affect ecological and evolutionary dynamics and forecasting^6,8,10–12^.

Here we consider only two sources of selective variation: genetic variation (additive and non-additive), and variance in phenotypic plasticity (we ignore neutral genetic variation). Hereafter we call these, respectively, genotypic (G) variation and genotype-by-environment (GxE) variation. We also consider three sources of non-selective variation. Firstly, environmental variation that affects every individual’s vital rates (often defined as environmental stochasticity). Secondly, non-selective demographic variation, stochastic demographic processes that determine the reproduction and survival of an individual and are selectively neutral^7,9,10,12,13^. Finally, we consider non-selective variation that is true noise due to, e.g., non-selective measurement error, data processing error, or hidden and/or uncontrolled experimental conditions.

To decompose the five sources of variability among individuals in survival and reproduction in nature, an ideal experiment would track many individuals with known genotypes over their lives, with the environment perfectly known, and no measurement or processing errors. Of course, such ideal conditions cannot exist. To approach such conditions an increasing number of studies have followed marked and genotyped (pedigree) individuals in the wild^14–18^. Many of such studies lack the large numbers of individuals needed to reveal causation beyond interpreting a simple correlation between the environment, the genotype, and an individual fitness component^19^. The limited population sizes and small numbers within pedigrees or genotypes leads to biased correlations and variance decomposition^20^.

Here, as an illustrative example, we analyze data from an experiment in which large numbers of seedlings of the common ribwort plantain, *Plantago lanceolata,* from multiple experimental crosses, were planted in a multiple cohort design and individuals were followed until death (Appendix Sketch). To this end we have many individuals of the same genotype (known crosses) that experience similar environments among years. To decompose the variability in survival and reproduction among individuals we used a matrix model approach to analyze genotype-environment specific population dynamics. For each year-sire combination, we constructed a stage-structured matrix model and computed macroscopic demographic parameters (population growth rate, generation time, average lifetime reproduction, life expectancy), and the variance in lifespan and lifetime reproduction. We quantified genotypic variation (G) in lifespan and reproduction by estimating the variance among the 16 sires used for the crosses. We computed the contribution of the genotype by environment interaction (GxE) by the variance among year-sire combinations. We used the variation among the six years to determine the non-selective environmental variation (E), and the variation among 17 spatial blocks to determine small-scale spatial environmental variation. The non-selective demographic variation was estimated directly from the matrix model for any year-sire class that went beyond the expected (mean) growth^10,21^. We assumed that this additional variance in growth was directly or indirectly related to the differences in survival and reproduction among individuals. Previous approaches have inferred the non-selective demographic variation from the residual variances of linear models as for instance done in most quantitative genetic models. Our approach differs in directly estimating the non-selective demographic variation in survival and reproduction from the models, which was possible due to the stochastic nature of these stage-structured models^10,21^. In applying this approach, we reached our aim which was to disentangle the genetic, environmental and demographic causes of variability among individuals in reproduction and survival and thereby identify drivers of population dynamics.

We found that, despite substantial fluctuation in survival and reproduction among years, the non-selective demographic (i.e. stochastic) variation explained substantially more variation in survival and reproduction compared to the joint effect of the environment (non-selective environmental variation), the genes (G), and their interactions (GxE). We use these results to illustrate the influence that chance among individuals has on ecological and evolutionary population dynamics. We briefly discuss the advantages of our approach over alternative approaches, such as mixed effect models, that do not directly estimate or link to such demographic parameters. Our results highlight the challenge of distinguishing between adaptive genetic variability and neutral variation in evolutionary ecology, population biology, and demographic studies in the field.

## Results

The overall population growth rate λ was high, at 7.61, the cohort generation time T_c_ was 2.78 years, the reproductive value R_0_ was 20.0 expected seedlings recruited, the average lifespan was 2.67 years, the variance in lifespan was 6.32 years^2^, the average lifetime reproduction was 0.421 inflorescences (reproductive stalks), and the variance in reproduction was 1.42 inflorescences^2^. Our findings are consistent with previous studies with this data^17,22–24^.

### Variance decomposition of lifespan and reproduction

Contributions to the total variance in lifespan and reproduction among individuals showed that only a small fraction (~ 0.5–1%) is explained by additive genetic (sire [G]) effects (Table 1). Non-selective environmental variability among years (E) explained little variance (2.5–4.6%) in reproduction and ~ 25% of the variance in lifespan. A small fraction of the variation is explained by the genotype-by-environment (year-sire [G*E]) interactions (4.6% to 6.7%) (see also^24^). The largest fraction of the variance is associated with unexplained variability in size within years and with sires that goes beyond expected size-specific reproduction or survival. We argue that most of the variation in survival and reproduction was caused by non-selective demographic processes.

We initially focused on variability among years, but geographic variation, even at a small scale, might influence survival and/or reproduction. To test for small-scale spatial variation, we included the 17 blocks as a random variable when estimating the regressions for each model. Counter to expectations, variability for lifespan slightly increased when accounting for spatial block differences (Table 1). Variability in reproduction doubled when accounting for block differences compared to the initial model with no spatial component (Table 1). Further, and again counter to expectations, there was no increase in genetic (sire) or environmental (among year) contribution, after correcting for spatial environmental differences (Table 1). We also evaluated small-scale microsite variability using smaller units of 64 plots, but our results (not shown) were no different from the analysis with the 17 larger blocks.

**Table 1:** Variance and the variance decomposition into genetics (sire), environment (variability among years), gene x environment interactions, and non-selective stochastic demographic variation for lifespan, and reproduction for two sets of models, (i) overall model without accounting for any spatial environmental variability, (ii) accounting for 17 blocks of the study field.

	Overall model		Block corrected	
	Lifespan (years)	Reprod. (inflorescence)	Lifespan(years)	Reprod. (inflorescence)
Absolute variances	6.32	1.42	6.87	2.83
**Fractions of the variance decomposition**				
Genetics (sire)	0.008	0.011	0.0075	0.0045
Environment (year)	0.245	0.046	0.250	0.025
GxE	0.067	0.064	0.065	0.046
Stochastic	0.680	0.878	0.678	0.925

**Figure 1:**
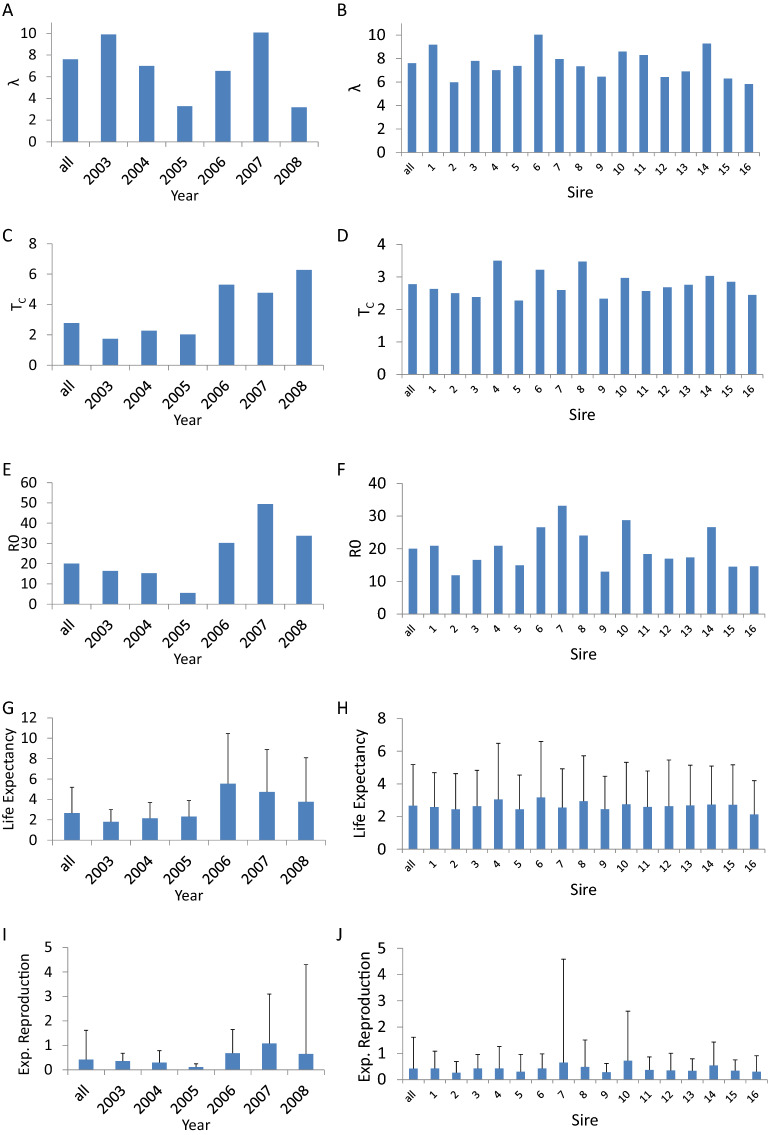
Differences in population growth rate λ (**A**, **B**), cohort generation time T_C_ (**C**, **D**), net reproductive rate R_0_ [expected number of seedlings recruited] (**E**, **F**), life expectancy (**G**, **H**), and expected reproduction [expected number of inflorescences] (**I**, **J**) among years (**A**, **C**, **E**, **G**, **I**) and sires (**B**, **D**, **F**, **H**, **J**). The most left bar depicts the value across all years or sires, weighted by the individuals within each year-sire combination. For life expectancy and reproduction (**G**, **H**, **I**, **J**) we plotted the mean + Stdev. This standard deviation comes from unexplained variability in size within years and sires that is not related to either reproduction or survival, we argue here that this variability is largely due to non-selective variation among individuals sharing the same sire and environment (year). See also Table 2, Appendix S1^10,21^.

Our study clearly shows the importance of variability among individuals within each genotype-by-environment (sire-by-year) class, which is a new finding from these data. These results show how key demographic parameters vary among models parameterized for different years and genotype combinations. Specifically, expected population growth rate λ varied substantially among years (Fig. 1A); generation time, T_c_, was relatively short in the first three years compared to the remaining years of the study (Fig. 1C), as was net reproductive rate R_0_ (Fig. 1E), average lifespan (Fig. 1G), and expected reproduction (Fig. 1I). Additionally, offspring of different sires differed substantially in population growth rate λ (Fig. 1B), generation time, T_c_, differed slightly less among sires (Fig. 1D), and net reproductive rate, R_0_ varied tremendously among sires (Fig. 1F). Lifespan varied to a lesser degree (Fig. 1H) compared to expected reproduction (Fig. 1J).

## Discussion

We have used a plant dataset to illustrate the importance of non-selective demographic processes on population dynamics. We analyzed fitness components and overall fitness, λ, by using experimental data to parameterize stage-structured matrix population models for each distinct gene-environment combination. Our approach allowed us to examine genetic, environmental and stochastic variation in survival and reproduction. We found that selective variation, genetic (G) and genotype-environmental (GxE) variation makes a small contribution to total variance in survival and reproduction, as does non-selective environmental variation, whereas non-selective demographic variation is very large. The design and richness of this data set allowed us to extend previous analytical approaches^25,26^. The important features of the data that made our analyses possible included the detailed demographic information on many marked and mapped individuals stemming from a small number of genotypes (sires). The precise marking and mapping resulted in negligible detection and measurement errors, for leaf counts, inflorescence counts and survival, for each individual plant. Additionally, the experimental design excluded within-species density dependence, because the spacing among the planted individuals was large enough to avoid direct competition. Natural recruitment from the experimental plants was not allowed thus there was no increase in within species density over time.

To critically examine our claim that the dominant source of variance in fitness components is non-selective demographic variability, we analyzed two alternative sources of variation, the micro-site environment and genetics. With respect to the environment, the randomized block planting design was used to minimize small-scale environmental influences in a field setting where spatial environmental differences among individuals cannot be completely avoided. If such differences contributed to major variation among individuals, we would expect non-selective stochastic variability in fitness components to decrease substantially after we accounted for small scale geographic differences (blocks) in our models, while the relative genetic (sire) and among-year environmental contributions would increase. But accounting for the blocks, or even for smaller spatial scale environmental differences of 64 sections of the 70 m * 35 m field site (results not shown), did not reduce the overall variability in fitness components as expected. Moreover, the relative genetic and among year environmental contributions did not systematically increase, as expected (Table 1). We cannot completely exclude that even finer scaled environmental differences might influence survival and reproduction among individuals within this population but without additional experimentation this source of variation cannot be clearly identified. Moreover, non-selective demographic processes do contribute much of the variability in fitness components.

**Table 2:** Notation and equations.

Description	Equation	Notes
	${e}_{t}$	Vector of zeros with a 1 at position t
	${e}^{T}$	Vector of ones, superscript $T$ denote transpose
Identity matrix	$\mathbf{I}$	
Stage transition matrix	$\mathbf{P}$	Includes survival and stage changes
Stage duration matrix	$\mathbf{N}={(\mathbf{I}-\mathbf{P})}^{-1}$	Elements quantify the expected time spent in each stage conditional on the birth stage
Mean Lifespan	$exL={e}^{T}*\mathbf{I}*\mathbf{N}*{e}_{t}$	
	$$exLsq={e}^{T}*\mathbf{I}*\left(2\mathbf{N}-\mathbf{I}\right)*\mathbf{I}*\mathbf{N}*{e}_{t}$$	
Variance in lifespan	$VarL=exLsq-{(exL)}^{2}$	
Fertility matrix	$\mathbf{F}$	
	$\widehat{\mathbf{F}}=diag(\mathbf{F})$	Diagonal elements of fertility matrix
Expected reproduction	$exR={{e}_{t}}^{T}*\mathbf{F}*\mathbf{N}*{e}_{t}$	
	$$exRsq={{e}_{t}}^{T}*\mathbf{F}*\left(2\mathbf{N}-\mathbf{I}\right)*\widehat{\mathbf{F}}*\mathbf{N}*{e}_{t}$$	
Variance in reproduction	$VarexR=exRsq-{(exR)}^{2}$	
Population growth rate	λ = dominant Eigenvalue of $\mathbf{X}$, with population projection matrix $\mathbf{X}=(\mathbf{F}+\mathbf{P})$	
Cohort generation matrix	${\mathbf{A}}_{\mathbf{c}}=\mathbf{F}*\mathbf{N}$	
Net reproductive rate	${R}_{0}=dominant Eigenvalue of {\mathbf{A}}_{\mathbf{c}}$	
Right eigenvector corresponding to dominant eigenvalue of ${\mathbf{A}}_{\mathbf{c}}$	$c\upsilon$, normalized so to sum of components = 1	
Left eigenvector corresponding to dominant eigenvalue of ${\mathbf{A}}_{\mathbf{c}}$	$d\upsilon$, normalized so to ($d\upsilon ,c\upsilon )=1$	
Cohort generation time	${T}_{c}=({d\upsilon }^{T}*\mathbf{N}*c\upsilon )/({d\upsilon }^{T}*c\upsilon )$	

Details and proofs of equations are found elsewhere^21,73^.

Genetic variability may also contribute to fitness variability among individuals in this non-clonal species. To include this source of variation, our primary analyses accounted for the effects of the dam. Another approach is to reverse the genetic focus by swapping dams and sires, i.e., assess dam effect while correcting for sires as random effects. When we did this analysis, our results do not change qualitatively (analysis not shown). Our low estimates of additive genetic (sire) and gene-by-environment contributions suggest that additional genetic differences among individuals stemming from the same sire cannot account for much of the unexplained variability in survival and reproduction.

Our models estimated a very high overall population growth rate λ of 7.42, which is obviously unrealistic for any natural population (see similar estimates from a deterministic life table response experiment (LTRE) analysis of the same experimental population in Shefferson and Roach (2012)). Because of the high accuracy in tracking individuals, their survival and the number of inflorescences, we believe this high estimate comes from ignoring losses in the transition from seed to established seedling. Seed predation^27^ and intraspecific density regulation were excluded in the experiments and would lower recruitment into the population and population growth. Irrespective of the exact cause for the high estimate of population growth rate, our main result that decomposed variability into genetic, environmental, and stochastic components should not be qualitatively affected (Online Appendix Fig. S3, Table S1).

To highlight how influential the within year-sire variation in survival and reproduction are for evolutionary population dynamics we an closely examine some specific findings. Variability in the population growth rate λ among sires (Fig. 1B) is less extreme compared to the variability among environments (years; see Fig. 1A). The sires that would be expected to go extinct first (Fig. 1B, sire 2, 9, & 16), are the ones that have low reproductive rates (Fig. 1F) and tend to have fast life histories^28^, i.e. low cohort generation time (Fig. 1D). However, these patterns require closer examination: Sire 7 has the highest expected reproduction, with a mean lifespan and a generation time that does not differ much from the population average. One would therefore expect sire 7 to have high fitness (λ)^21^, but this expectation does not hold because reproduction varies substantially within the sires’ offspring (Fig. 1J). These results caution us not to interpret demographic parameters in isolation, at least when making evolutionary ecological predictions.

Despite the dominating influence of non-selective demographic effects, environmental variation among years was strong. During the course of the six years analyzed in this study, there were three years of high mortality and decreased λ, which suggests stressful environmental conditions (2003–2005) (Roach et al. 2009)(Fig. 1A). Generation time and net reproductive rate R_0_ indicate fast life histories with short expected lives and low expected lifetime reproduction (Fig. 1). Interestingly, there were no carryover effects of the stress in these years, with respect to survival and reproduction (see also little shift in size distribution across the years Online Appendix Fig. S2). This lack of carryover is particularly interesting given that small, and in particular small, old, individuals died at high rates during this stressful time^23^.

Our estimates of the relative size of additive genetic variation might be considered small, but a low additive genetic contribution to the total variability in survival and reproduction among individuals is not surprising from a population genetics perspective or basic evolutionary theory^29–31^. Indeed, many studies of natural populations find low estimates of heritabilities for fitness components^32–37^. Given low heritabilities, and the sample sizes (per genotype) typically found in ecological studies, detecting any evolutionary change within the time scale of such studies will be challenging^10^. Our low estimates of additive genetic variation do not mean that the variability is evolutionary unimportant but rather, that selective changes will be slow and genetic drift enhanced, particularly in populations with long or complex life cycles^10,19,38,39^.

Our estimates of the amount of non-selective demographic variation are consistent with what has been found for fitness components in individuals of model organisms raised in the lab under highly controlled conditions. Even among isogenic individuals under lab conditions the coefficient of variation (CV) of the stochastic demographic component ranges between 0.24 to 1.33 in lifespan (*Caenorhabditis elegans* 0.24–0.34^40,41^, *Caenorhabditis briggsae* 0.31–0.51^42^, *Saccharomyces cerevisiae* (0.37)^43^, *Escherichia coli* 0.4–0.6^44,45^). Less controlled studies that include individuals with more genetic variation do not differ much from these patterns in the CV for lifespan, for example in laboratory reared mice (0.19–0.71)^41^ or *Drosophila melanogaster* 5.98–13.48^46^. These values are in the range of the values we detect here: CV 0.96 for lifespan, and 3.97 for reproduction (non-block corrected estimates). Such estimates are also well within the range of variability in lifespan and reproduction of other natural populations, even though the decomposition into non-selective stochastic components is often not possible under less controlled conditions^7,13^.

The example we present here concerns one population of a perennial plant species but might be more general. With respect to growth form and demography, *Plantago lanceolata* is typical of all herbaceous plant species that includes perennials such as this species, and annual species. Unfortunately, very few demographic studies have been done with other plant species (but see^47^ for a review of life history trade-offs and senescence in other herbaceous plants). Beyond herbaceous species, the results in this study, are consistent with a long-term study with *Fumana procumbens*, a perennial dwarf shrub, that shows large environmental effects on age-and size-dependent survival and reproduction^48^.

The large variability in fitness components among individuals that cannot be explained by genetics or the environment remains a mystery if not considered in the context of non-selective stochasticity. Various approaches support such understanding and provide similar estimates on variance from a range of studies conducted in the field^18,49–51^. Analyses based on exceptionally deep pedigree and applying the principle of individual reproductive value, show that less than half of the variation among the individuals genetic contribution to the next generation, can be explained by individual lifetime fitness, comprising lifespan, lifetime reproductive success, and projected growth rate^18^. This estimate still biases stochastic contributions low, because selective and neutral genetic variation cannot be fully decomposed. Other studies support the idea that stochastic events play a major role at all levels, from stochastic gene expression, to the protein level, to the cell and organismal organization level, and these studies range from relatively simple organisms such as bacteria to complex ones such as mammals^52–58^. As population genetics has long recognized, such variability in individual fates means that stochastic effects are larger in small populations. The combined effect of the individual variability we discuss here and the actual population size is measured by the effective population size^31,59,60^.

Our approach differs from previous models that estimate non-selective demographic variability^61,62^ by including stages and not just age. In previous models, demographic variability has been estimated as the variance and covariance in survival and fertility within an age class, but these models do not provide a mechanism to correlate performance across ages because age classes are assumed to be independent. Further, these models do not consider cohorts, which then makes it impossible to compute life history traits such as an individuals’ age at death, or lifetime reproductive success. This limitation thus makes alternative models less applicable for assessing life history tradeoffs, such as the fundamental tradeoff between survival and reproduction^63^. Additionally, previous models have included approximations for stochastic dynamics (transient dynamics), whereas the models presented here are deterministic. Excluding stage dynamics (here size as stage) would lead to very different estimates of non-selective demographic variability, because in our model the stage dynamic is one of the main processes generating variance and is crucial for the computation of the correlation between growth, survival and reproduction within and among ages (the latter through the Markovian structure of the model). Such stage dynamics thus allow us to compute the non-selective stochasticity we were mainly concerned with, and thereby account for the importance of quantitative trait dynamics for life histories. Our focus on individual measures also recognizes the central role that individuals play in demographic and population changes, including fundamental tradeoffs and covariances between longevity and lifetime reproduction^1,63^. Here, we have shown that variation driven by stochastic demographic processes can be precisely quantified by a structured matrix model, and this approach provides us with a better understanding of population dynamic processes, including the effect of stochastic events on population growth and other fitness related demographic parameters.

In ecology and conservation biology, the role of stochastic demographic processes has been mostly investigated for population extinction processes rather than evolutionary ecological processes^2,64,65^, but see more recent work^18,50,61,66^. Ecological models provide insight into the effects of stochastic environmental variation on vital rates, but surmise that stochastic demographic variation is of little importance because, as long as the populations are not very small, extinction is not influenced by such processes^2^. However, in the context of evolutionary dynamics, the adaptive potential to respond to climate change can be substantially influenced by large amounts of stochastic demographic variation and may in the long run influence extinction even in large populations^10,38^. The data used here is a single illustrative example that shows that large within year-sire variation in reproduction does not lead to high expected growth rates of these sires. It highlights, that even in large populations, evolutionary population dynamics, and consequently long run extinction, can be influenced by such neutral variation.

## Conclusions

Historically, unexplained variation (residual error) has often been interpreted as a lack of knowledge of underlying causes, measurement error, and/or limited control of the conditions under which experiments are conducted. Recent studies have been devoted to determining the impact of stochastic events on variation at the molecular, cellular and organismal level. At each of these levels substantial evidence suggests that stochasticity plays an important role^18,44,49,51–57,67^. In some circumstances the cause of the “stochastic” outcome can be tracked down to a mechanistic cause at a lower level, but this mechanistic cause may be triggered by other stochastic events. If such cascading stochastic (snowball) events play a major role in determining demographic variation, then variability in fitness components must be seen as neutral and not driven by the environmental or genetic variability among individuals. Controversial discussions about neutral theories, in molecular evolution or community ecology, also highlight the idea that these processes are ubiquitous, at many different levels, but are not easy to quantify^68–70^.

We have shown that large amounts of variability in fitness components among individuals in this study are likely due to stochastic demographic processes and such neutral variability has significant effects on population dynamics and demographic parameters. Our understanding of this type of variability and its impact on the evolution of phenotypic variability is limited and we call for more attention to and focus on understanding such variation. These neutral processes have ecological and evolutionary consequences, but neither our current theories nor our empirical understanding are sufficient to explain their evolution and maintenance.

## Materials and methods

*Plantago lanceolata* is a widely distributed short-lived perennial herb. In the experimental field site, located at the Shadwell Preserve of the Jefferson Monticello Foundation, Shadwell, Virginia, USA, this species maintains a basal rosette of 1 to ~ 240 leaves (mean 12.5 ± 11.53 SD). It germinates both fall and spring and remains green all year, thus individuals can be easily followed for size (number of leaves) and survival throughout the year. Seeds for this study were produced from crosses with parental individuals that had been randomly collected from the 70 m * 35 m field site. Parental genotypes were crossed using a modified North Carolina II design^71^. Here we used crosses that consisted of four sires crossed to each of two dams resulting in eight sire-dam combinations and 200 offspring from each. This was repeated for five unique sets of sires and dams (20 sires, 10 dams), 40 sire-dam combinations and 8000 individual offspring. In the analysis reported here, we used individuals from the 32 crosses with the largest replication^22^. This design was used for cohorts 1 and 2 (planted in years 2000 and 2001) and one-half of the total number of individuals per cross was used for cohorts 3 and 4 (both planted in year 2002, spring and fall respectively, and for this analysis these cohorts were classified in the same age class). All cohorts had the same genetic structure when initially planted. Further details of the planting design and protocol are reported elsewhere^22^. The analysis reported here began with the data collected in 2003 when 6913 individuals from these 32 crosses had survived. At this time, the number of individuals per cross, across cohorts, ranged from 283 to 376, and spatially these individuals were distributed across 17 randomized blocks and 64 plots. The matrix models constructed here use size (number of leaves) and survival data (censused and recorded in May/June each year) from 2003–2008. In total, there were 14,082 events where size and survival in consecutive years was known. Data collected prior to 2003 was excluded because not all cohorts had been established before this period.

### Size structured matrix models

The analyses are based on discrete time (Markovian) size-structured matrix population models with number of leaves (size) as the stage character and the number of reproductive stalks (inflorescences) as a measure of reproduction. Vital rates (growth, survival and fertility), were estimated using regression models (see below) as is normally done for integral projection models (IPM)^72^, except that here we used the regressions to parameterize discrete size structured matrix models^26^. The stage structure consisted of 100 size classes (one for each number of leaves between 1 and 100 +).

For each year-sire combination (6 years [2003–2008] and 16 sires, each sire crossed with two dams) we fit one model with size as the stage characteristic (Online Appendix Fig. S1). From each of these year-sire specific models we directly computed: population growth rate λ, generation time T_c_, net reproductive rate R_0_ (number of seedlings produced), expected reproduction (expected number of inflorescences produced) and variance in reproduction (variance in number of inflorescences produced) among year-sire individuals within the year-sire combination (non-selective demographic variation in reproduction), and life expectancy and variance in lifespan among year-sire individuals (non-selective demographic variation in survival within the year-sire combination)^21,73^ (Table 2).

### Data analysis

For each model (i.e., year-sire combination), four regression functions were fit describing the relationship between the individual’s a) current size and survival, i.e. size-specific survival function; b) current size and size at time t + 1, i.e. size-specific expected growth and the variance in size-specific growth among individuals; c) current size and reproduction; i.e. the size-specific probability of producing at least one inflorescence; and d) current size and number of inflorescences given that at least one inflorescence was produced, i.e. size-specific production of inflorescences (Online Appendix Fig. S1). In the final two years (2007 and 2008) one sire had too few surviving individuals (N = 24 and N = 9) to fit meaningful functions; we thus excluded these two years for this sire (# 16), which left us with 94 models (16 sires * 6 focal years; equals 96 minus the two years for sire #16). To estimate the non-selective environmental variance across all years and the additive genetic variance (G) across all sires, we used the demographic parameters from these 94 year-sire specific models weighted according to the number of individuals in each year-sire combination, i.e. year-sire combinations that had more individuals contributed more to the weighted variances across all years or sires than those with fewer individuals.

All functions were fit to square root transformed size measures to improve normality in residuals. For the survival and the reproduction functions, a binomial model with a linear and quadratic term for size was used (see example Fig. S1a and c). A Poisson distributed model structure with a linear and quadratic term for size was used for the number of inflorescences (see example Fig. S1d), and for the growth function a Gaussian linear model was used (see example Fig. S1b). It should be noted that around these size-specific functions there is variance in the realized individual outcomes and among the year-sire specific functions. It is these two sources of variance that are core to our approach to decompose variance contributions^10^. Because of the limited number of very large individuals, we binned all individuals ≥ 100 leaves (per year-sire combination) into one size class and binned all individuals with ≥ 30 inflorescences (per year-sire combination) into one reproduction class. When estimating function parameters, we included the dams as a random (intercept) effect to account for potential differences among dams.

To build full population matrixes ${\varvec{X}}gy$, for sire $(g)$ in year $(y)$, we made assumptions regarding recruitment. The field experiment did not allow direct recruitment of seedlings into the study site, thus did not have sire $(g)$ specific recruitment rates ${x}_{yg}(k,z)$ where an individual of size $(z)$ in year $\left(y\right)$ contributes to the recruited individuals at size (stage) $k$ at time t + 1. For recruitment we used probability estimates from a separate study of seeds planted directly into the same field (Shefferson & Roach 2012). The probability of seedling establishment (0.1035) was computed from the germination rate of 0.69 and the seed to seedling survival rate of 0.15 (see robustness to assumptions about seedling establishment Online Appendix Table S1, Fig. S3). We estimated the age-specific fecundity as the number of inflorescence spikes produced by an individual. As we did not have exact seed numbers for each inflorescence, except for a small fraction of the plants included in this analysis, additional variation in seed mass and seed number across inflorescences could occur (see below for year specific estimates of variance in seed numbers). Our method to estimate fecundity from inflorescence numbers has been used for several other earlier studies with this species and it has a high correlation with total seed number. To account for differences among years in number of seeds produced per inflorescence, we took the mean number of seeds counted from approximately 150 randomly selected inflorescences each year. Mean number of seeds per inflorescence (± 1 SE): year 2003, 70.72 ± 2.83; year 2004, 52.83 ± 2.69; year 2005, 42.5 ± 2.43; year 2006, 42.07 ± 2.28; year 2007, 50.86 ± 1.82; year 2008, 27.84 ± 2.09. For the seed to seedling size distribution, we assumed that these were equal across years and sires.

To test for potential effects of small-scale environmental differences throughout the 70 m * 35 m experimental field we corrected for variance among 17 blocks, small-scale geographic units across the experimental field. In this set of analyses we included one additional random effect, the block (N = 17), for estimating the size dependent regression functions for each model (year-sire combination). Comparing the two sets of analyses (with and without correcting for variance associated to the blocks) provided insights on micro-climatic and small-scale environmental effects throughout the experimental field.

The four regression function parameters (Fig. S1) together with the number of seeds per inflorescence, the probability of seedling establishment, and the seedling size distribution were used to compute two 100*100 matrixes (100 size classes) for each year-sire combination. One matrix, the transition matrix $P$ (Table 2) contained survival rates and expected growth (change in number of leaves) and the variance around that expected growth, i.e. the probability of transitioning from a given size at time *t* to size at time *t* + *1*. The column sums of this matrix *P* are smaller than 1 and are equal to the survival rates of individuals at a given size at time *t*. The other matrix, the fertility matrix $F$ (Table 2), contained size-specific reproduction and recruitment rates, i.e. the probability of reproducing inflorescences with a yearly specific number of seeds, that then yields the recruitment of seedlings into the population depending on the current size at time *t*. We limited the maximum survival probability of any size to 0.95 (the results are robust to this assumption). The computation of the functions and the matrixes were done in program R^74^.

Each pair of matrices was used to compute: (i) population growth rate λ, (ii) cohort generation time, *T*_*c*_, (iii) net reproductive rate *R*_0_ (expected number of seedlings recruited), (iv) mean lifespan, (v) variance in lifespan, (vi) mean reproduction (number of inflorescences), and (vii) variance in reproduction for each year-sire combination^21,73^ (Table 2). The stochastic properties of these matrix models allowed us to directly compute the expected variances in survival and reproduction. The stage duration matrix^73^, *N*, (a.k.a. the fundamental matrix, see Caswell (2009)), was used to estimate the mean and variance in lifespan and reproduction for each year-sire combination^10^. The elements of this matrix quantify the expected time an individual spends in each stage conditional on the individual’s birth stage (Table 2)^10,21,73^.

Our models are based on year and sire, not age or cohort, because we found that size-distributions did not shift along with age-structure during the study (Online Appendix Fig. S2). Models that included only age and year, or age and sires (not shown) did not lead to qualitatively different results. Previous studies using this data^17,22–24^ revealed that survival dropped significantly in 2003 for all cohorts or ages^22^. A model comprising age-and-year-and-sire combinations could not be fit here because of lack of sample size; moreover, such a complex model would be difficult to interpret biologically.

We use matrix models to decompose the variability in survival and reproduction among individuals, which is in contrast to mixed effect models, GLMs, ANOVA, or similar approaches that are often used for a decomposition of the total variances, into the genetics, the environment (year), G x E, and the unexplained residual components^75^. These latter approaches produce biased estimates for various reasons. First, when estimates of genetic and environmental variance are based on low numbers of individuals within the same year or individuals that are closely related, such models are anticonservative and underestimate the stochastic component^20^. Our analyses are less affected by such effects because of larger within year-sire numbers. Second, for a multiple cohort study such as ours with fewer cohorts than age classes, a mixed effect model would underestimate variance in mortality because of the limited age-structure in a given year, which is a general problem in many studies tracking known-aged marked individuals, whereas variance in reproduction would be more accurately estimated (see Online Appendix Table S2). Third, a multitude of mixed models can be fitted with results that are often highly sensitive to factor combinations (see Online Appendix Table S2).

Our matrix models include aspects of mixed effect models, but we do not include individual as random effects because our objective was to combine information about different life history traits and demographic rates. Our approach provides a direct link between survival probabilities and growth trajectories and we can easily combine different distributions (e.g. binomial for the probability to reproduce, Poisson for the number of inflorescences). The flexibility to combine information across models (e.g. survival and reproduction) is particularly challenging when using mixed effect models, though see developments in MCMCglmm^76^. Our approach made it possible to estimate, for each year-sire combination, a size structured matrix model that provides direct calculation of the non-selective demographic variation for each model^10,21,73^.

## Supplementary Information

Supplementary Information.
