# Supplementary material for: Quantifying the effect of genetic, environmental and individual demographic stochastic variability for population dynamics in Plantago lanceolata
Source: Sci Rep. 2021 Nov 30;11:23174. doi: 10.1038/s41598-021-02468-9 (PMC8633285; doi:10.1038/s41598-021-02468-9)
Supplement: Supplementary file 1 — Supplementary Information. [file 41598_2021_2468_MOESM1_ESM.docx]

Supplemental Information:

**Quantifying the effect of genetic, environmental and individual demographic stochastic variability for population dynamics in *Plantago lanceolata*.**

**Ulrich K. Steiner, Shripad Tuljapurkar & Deborah A. Roach**

**Sketch of Experimental design**

**
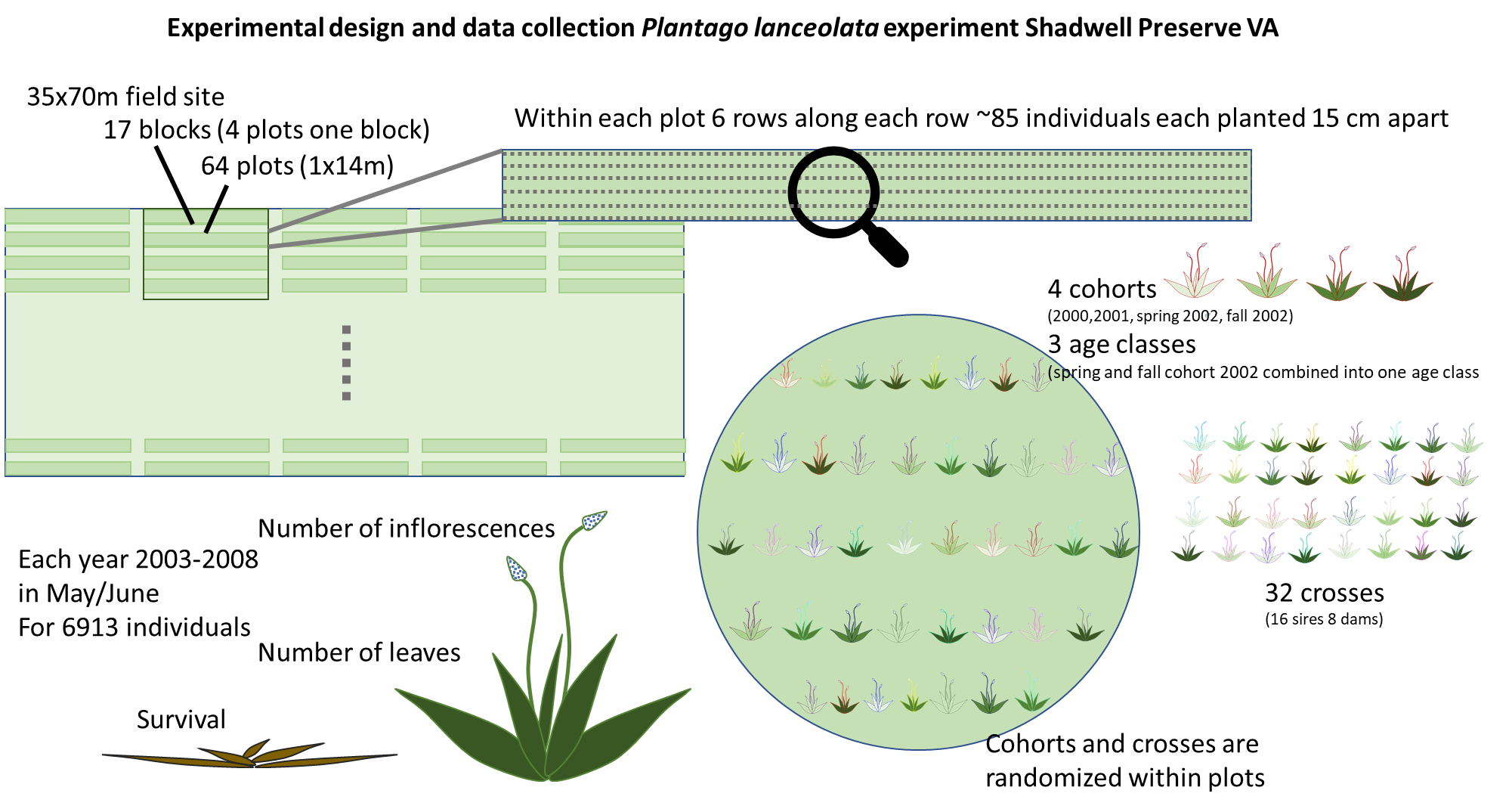
**

**Examples of regression functions fit to parameterize each (year-sire specific) mixed effect model**

| a)  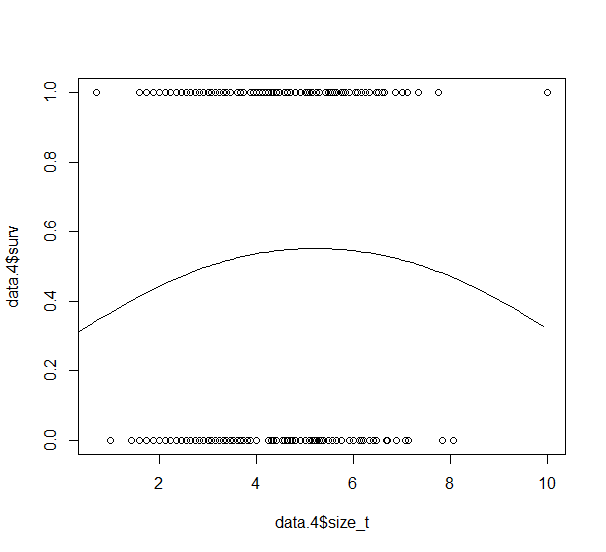  Survivaltt  Size at time t+1tt  Size at time ttt | b)  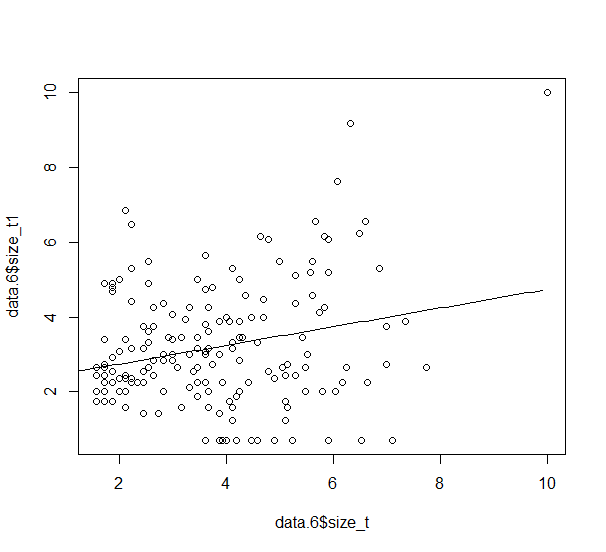  Size at time ttt |
| --- | --- |
| c)  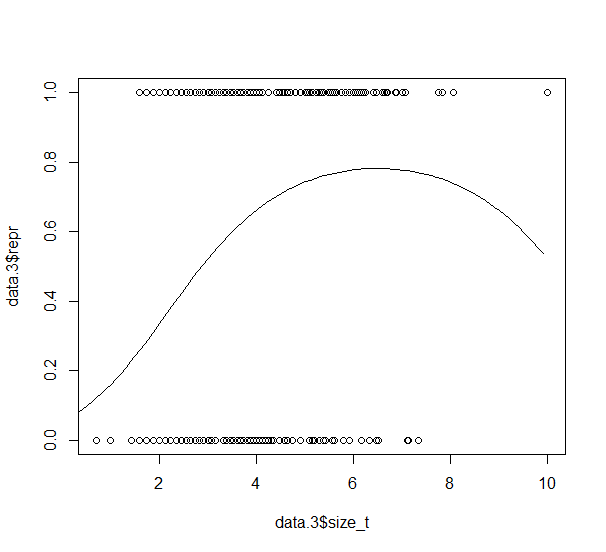  Inflorescencett  Reproducingtt  Size at time ttt | d)  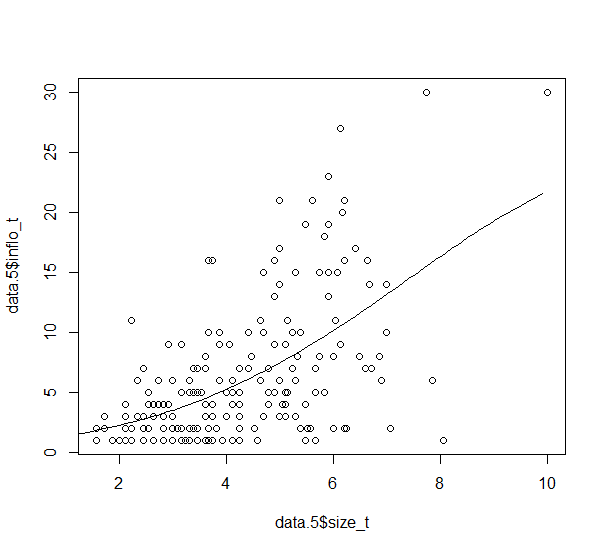  Size at time ttt |
| Fig. S1: Examples of four regression functions that were fit to the initial data from the marked individuals for each model. These regressions describe the relationship between the current individual’s size and a) survival, b) size at time t+1, c) reproduction, i.e. the probability of producing at least one inflorescence given the current size, and d) number of inflorescences given that at least one inflorescence was produced. For the growth function b), the variance was estimated and used for computing the matrixes and the sources of non-selective demographic variation in lifespan and reproduction that was not related to size. The data and functions shown here as examples, are for sire 1, in year 2003 (sire-year combination). These four functions were used to parameterize the size structured matrix model, i.e. the two 100*100 matrixes. One model was fit for each year-sire combination. All size measures were square root transformed. | |

**Variation in the size distribution over the different study years.**

The multiple cohort design of the experiment led to a shift in the age structure to older age classes during the study. Given that age and size might be partly correlated, the shift in the age structure might lead to a shift in the size structure with increasing years. Note that we excluded data collected prior to 2003 because not all cohorts had been established before this period. Fig. S2 illustrates that there was no systematic shift in size structure with shifting age structure in this study. There is variability in the size structure among years (mainly driven by the environmental variation), but a systematic shift in size distribution (expected towards larger sizes) during the experiment was not found. The first year, 2003 shows a distribution with relatively large individuals, 2004, 2005, and 2006, show distributions with relatively small individuals, while 2007 and 2008 again show slightly larger individuals.

| 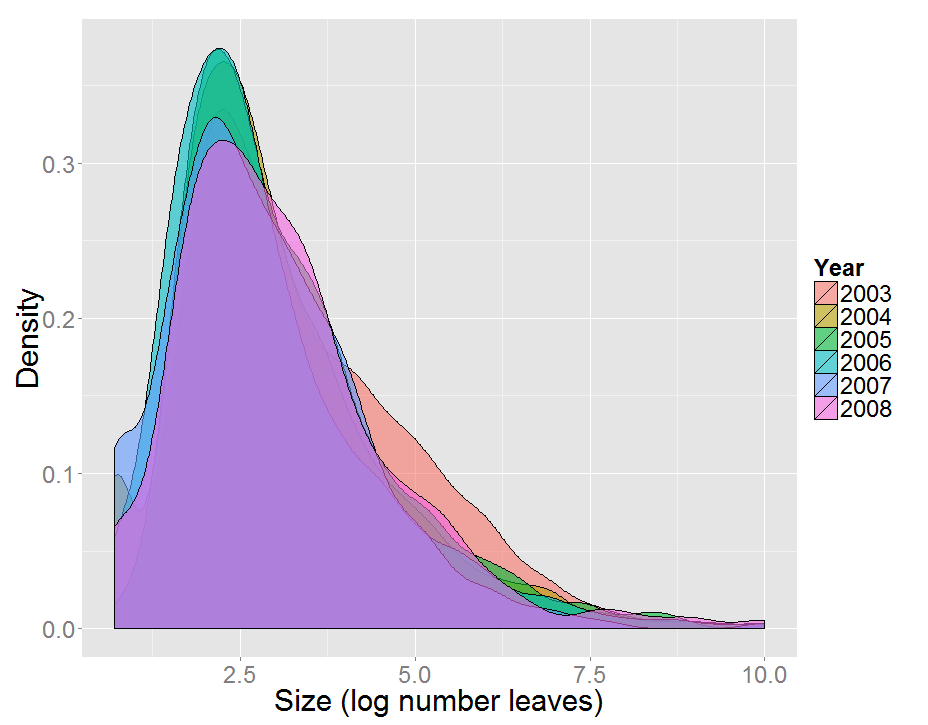 |
| --- |
| Fig. S2: Size distribution (log transformed, number of leaves) for the six study years, 2003-2008. |

**Robustness of results to assumptions about seedling establishment**

Table S1: Testing for robustness of the main result (stage-structured matrix models; Table 1 main text) to changes in the probability of seedling establishment. Here we only present results without accounting for any spatial environmental variability (i.e. no block effect included). In the original analysis (Table 1) seedling establishment was estimated at 0.1035, here we lowered the seedling establishment to 0.01035, hence assume a ten times lower germination rate (0.069) or seed to seedling survival rate (0.015) compared to the estimates used in the main test where seedling establishment was based on a separate study that planted seeds directly into the field (Shefferson & Roach 2012). All other parameters and estimation have been kept exactly the same.

|  | Model with lowered seedling establishment | | Original model (Table 1)  Seedling establishment (0.1035) | |
| --- | --- | --- | --- | --- |
|  | Lifespan (years) | Reprod. (inflorescence) | Lifespan (years) | Reprod. (inflorescence) |
| Absolute variances | 6.32 | 0.0285 | 6.32 | 1.42 |
| Fractions of the variance decomposition | | | | |
| Genetics (sire) | 0.008 | 0.007 | 0.008 | 0.011 |
| Environment (year) | 0.245 | 0.024 | 0.245 | 0.046 |
| GxE | 0.067 | 0.047 | 0.067 | 0.064 |
| Stochastic | 0.680 | 0.922 | 0.680 | 0.878 |

Reproduction does not influence survivorship and lifespan, for that the variance decomposition for that part of the analysis does not change. However, reduced seedling establishment lowered the absolute variance explained by reproduction, reduced the genetic, environmental and gene*environmental contribution to the total variance, and increased the stochastic component. The overall quantitative pattern remained similar. Further details of the effect on reduced seedling establishment are illustrated in Fig. S3.

| A  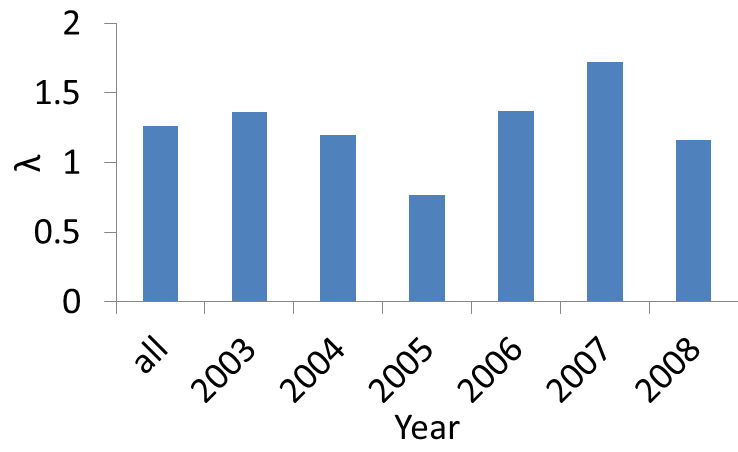 | B  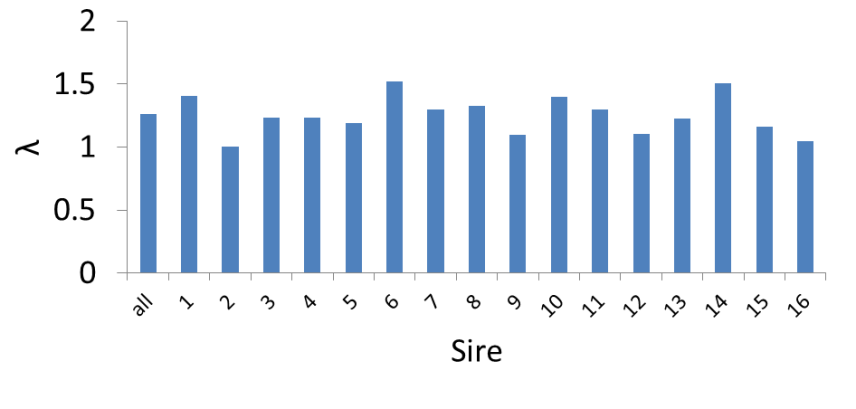 |
| --- | --- |
| C  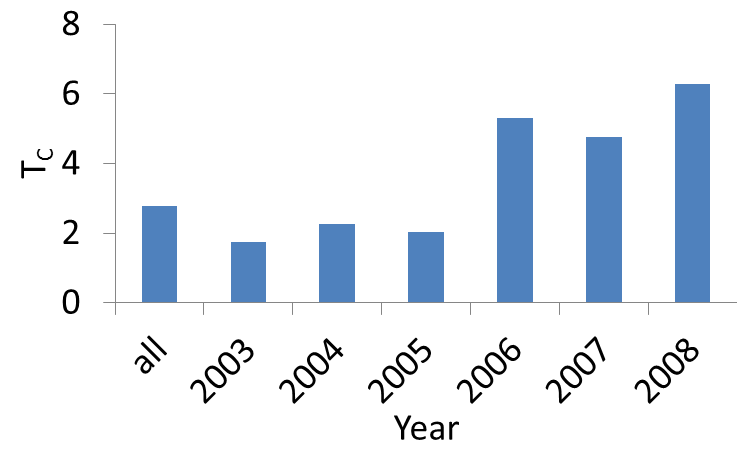 | D  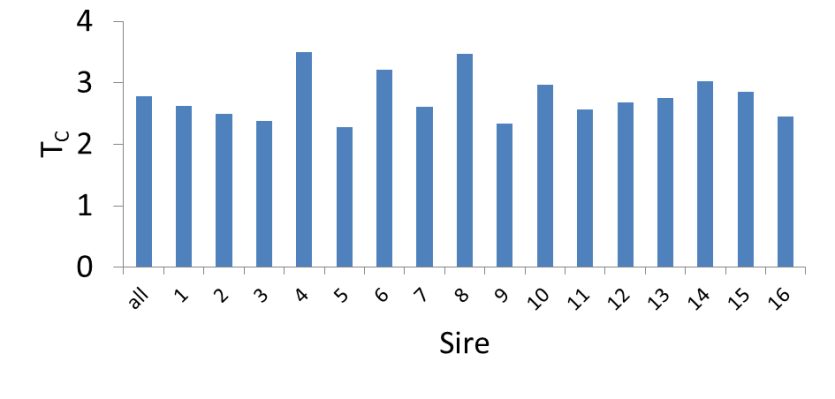 |
| E  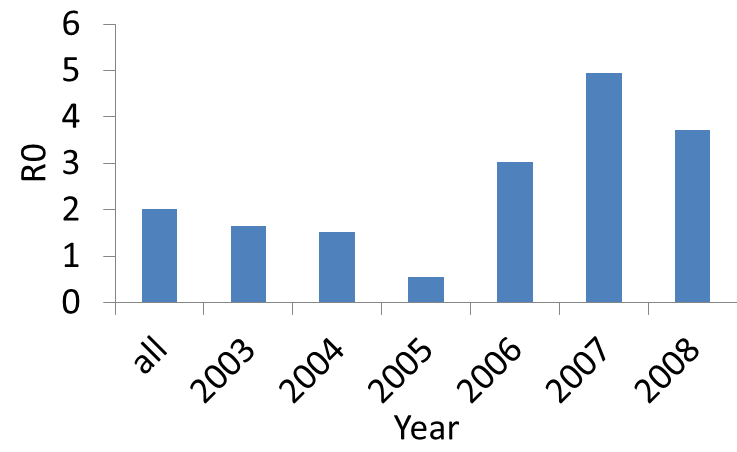 | F  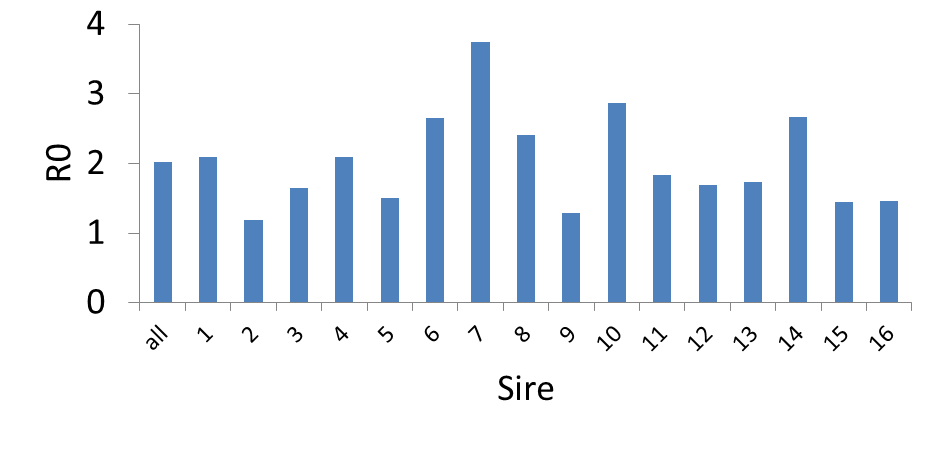 |
| G  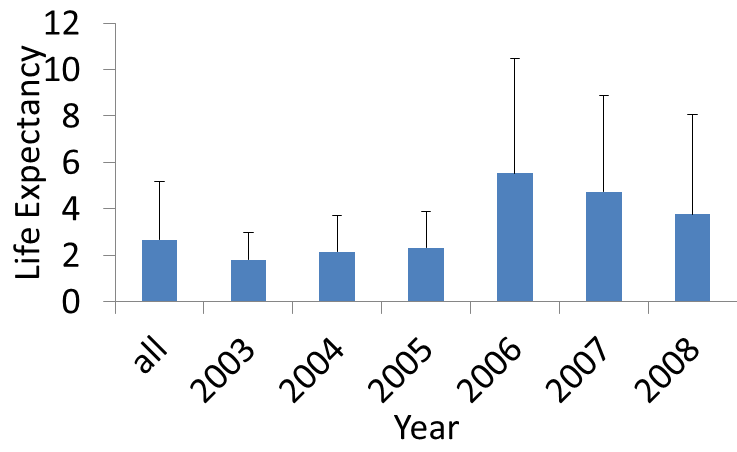 | H  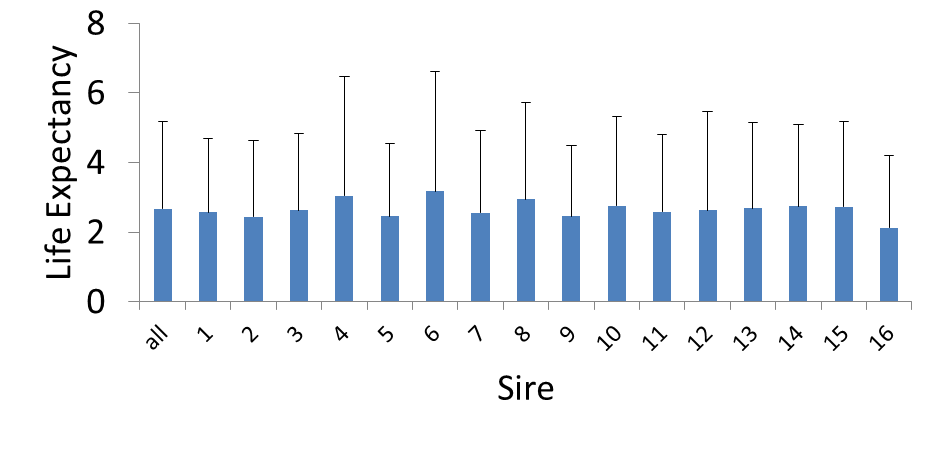 |
| I  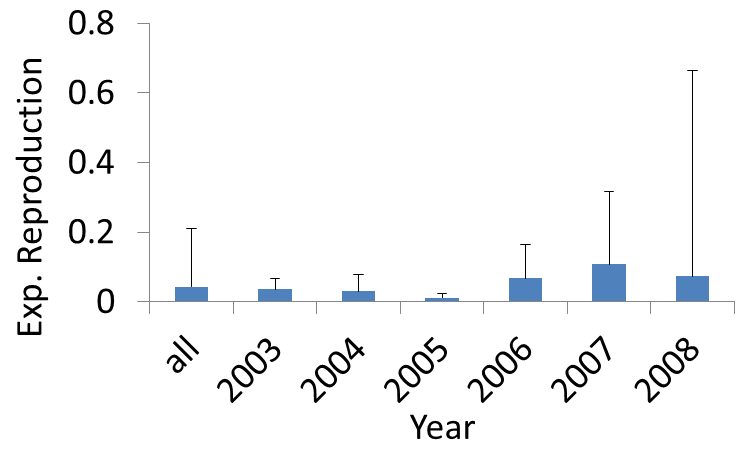 | J  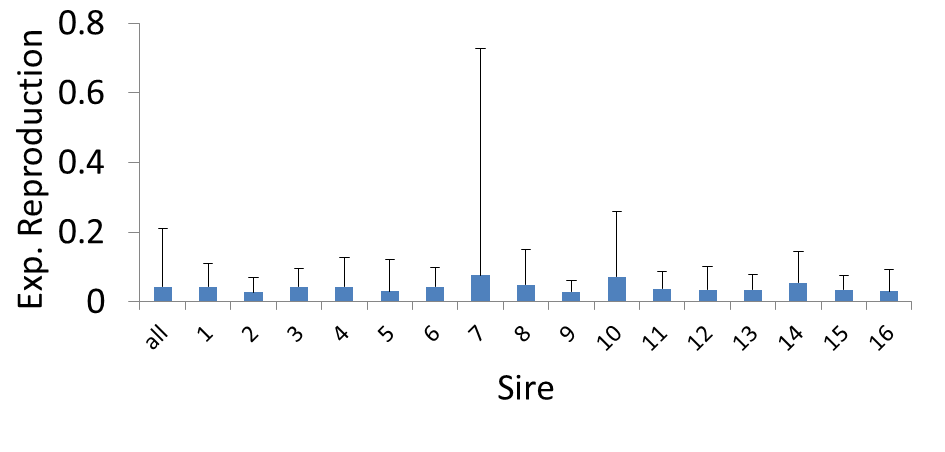 |
| Fig. S3: Differences in population growth rate λ (A, B), cohort generation time T_C_ (C, D), reproductive value R_0_ [number seedlings recruited] (E, F), life expectancy (G, H), and expected reproduction (I, J) among years (A, C, E, G, I) and sires (B, D, F, H, J). The most left bar depicts the value across all years or sires. For life expectancy and reproduction (G, H, I, J) we plotted the mean + Stdev. The seedling establishment is lower (0.01035) compared to the original analysis presented in the main text (Fig. 1), all else is kept constant. | |

**Generalized linear models and mixed effect models**

# Alternative approaches to our matrix model approach include GLMs and mixed effect models (GLMMs). We present a set of such models, even though they cannot be directly compared because they include different information. A multitude of linear models could be fit and our limited selection presented here is based on fitting models that include factors similar to the matrix model we have used in the main text. For the GLM and GLMMs we fit survival (binomial variable) and the number of inflorescences (Poisson error distribution) as the response variable respectively. For the mixed effect model we included sire and year, their interactions, as well as their interactions with (log)size as fixed effects, and included dam as random (intercept) effect. For a variance decomposition, we estimated the total variance explained by the fixed (and random effects) using the method developed by Nakagawa, & Schielzeth ^2^, function r.squaredGLMM, program R package {MuMIn}, that estimates a R^2^ value for all fixed effects together and the combined R^2^ of fixed and random effects. The residual variance (1- R^2^) is then interpreted as unexplained variance (non-selective demographic variance and non-selective noise). The ANOVA command in program R was then used to decompose the total variance explained by the fixed and random effects (R^2^) into the specific fixed effects (G, E, GxE). Similar sets of models were fit for the GLM’s except that the dam was included as an additional interacting effect instead of a random effect. Because the random dam effect was estimated to explain only a very small fraction of the variance, a third set of models were setup where we dropped the dam effect completely. Despite various convergence problems, we present the results in Table S2, but raise caution for interpretation. All models were fit in program R ^3^.

Table S2: Variance decomposition into genetics (sire), environment (variability among years), gene*environment interactions, and stochastic variation for survival, and reproduction (# inflorescences) of three sets of models, i) GLMM with dam as random (intercept) effect, ii) GLM with dam as interactive fixed effect, iii) GLM without any dam effect.

|  | Mixed effect model (GLMM) | | GLM (including interactions with dam as fixed effect) | | GLM (without any dam effect) | |
| --- | --- | --- | --- | --- | --- | --- |
|  | Survival | Reprod. (# inflorescence) | Survival | Reprod. (# inflorescence) | Survival | Reprod. (# inflorescence) |
| Fractions of the variance decomposition | | | | | | |
| Genetics (sire) | 0.109 | 0.3228 | 0.0056 | 0.012 | 0.037 | 0.280 |
| Environment (year) | 0.160 | 0.0437 | 0.0996 | 0.351 | 0.062 | 0.073 |
| GxE | 0.052 | 0.0144 | 0.0198 | 0.022 | 0.012 | 0.015 |
| Random effect (dam effect) | 0.001 | * | - | - | - | - |
| Residual error | 0.677 | 0.619* | 0.8750 | 0.615 | 0.889 | 0.632 |

*Convergence problem for r.squaredGLMM, hence residual variance estimated by getME(object, "devcomp"), R package lme4.

# Table S2 illustrates the difficulty in determining what factors should be included and how sensitive such models are to different parameter combinations. The variance in survival explained by the fixed effects drops dramatically when the dams are not included as a random effect, even though the random effect in the GLMM accounts only for a very negligible amount of variation (0.001). The variance decomposition in survival changes between GLM with and without dam as fixed (interactive) effects and the additive genetic contribution changes markedly from 0.56% to 3.7%. Residual variance for reproduction does not change as dramatically between GLMM and GLM’s, though the contributions of the different fixed effects changes extremely between GLMM and GLM’s. In the GLM with dam as fixed (interactive) effects, 35% of the total variance is explained by the environment (year) and only little is explained by the genetics (~1%). When we dropped the dam effect from the model or fit a GLMM with dam as random effect, most of the variance is explained by the genetics (28% and 32% respectively), and only relatively little (7.3% or 4,4%) is explained by the environment (year).

# It should be noted that the variance decomposition (Table S2) could not be directly compared to the matrix model results in the main text (Table 1), because the matrix models decomposed variance in longevity, including the size and growth functions, whereas in the linear models (GLMM & GLM) survival (binomial variable) was decomposed. For reproduction in the matrix models, we decomposed variance in reproduction that incorporated both probability of reproducing (binomial variable) as well as the number of inflorescences and their relationship to size, while in the linear models (GLMM & GLM, Table S2) only number of inflorescences was included.

1. Shefferson, R. P. & Roach, D. A. The triple helix of Plantago lanceolata: Genetics and the environment interact to determine population dynamics. *Ecology* **93**, 793–802 (2012).

2. Nakagawa, S. & Schielzeth, H. A general and simple method for obtaining R 2 from generalized linear mixed-effects models. *Methods Ecol. Evol.* **4**, 133–142 (2013).

3. R Development Core Team. R: A Language and Environment for Statistical Computing. (2012).
